# Cortisol and Daily Emotional Well-being: Weakened Associations With Age

**DOI:** 10.1093/geroni/igaf122.3273

**Published:** 2025-12-31

**Authors:** Rebecca Polk, Blake Ebright-Jones, Alexandra Freund, Derek Isaacowitz

**Affiliations:** Washington University in St. Louis, St. Louis, Missouri, United States; Washington University in St. Louis, St. Louis, Missouri, United States; University of Zurich, Zürich, Switzerland; Washington University in St. Louis, St. Louis, Missouri, United States

## Abstract

Older adults often report greater emotional well-being than younger adults despite declines in physical and cognitive functioning. Cortisol, a key stress hormone, may play an important role in these age-related differences in emotional well-being. As part of a larger study on aging, motivation and emotion in everyday life, we examined the relationship between daily cortisol output and emotional experience in 46 younger (M = 24.9 years, SD = 2.8; 36 women) and 51 older (M = 72.2 years, SD = 5.7; 26 women) adults. Participants provided daily saliva samples for cortisol analysis across six days, concurrent with ecological momentary assessments (EMA) capturing current valence and arousal (5-point scales) and affective choice (“If I could choose to engage with something RIGHT NOW, I would choose something that would make me feel…”). Area under the curve (AUC) was calculated to quantify total daily cortisol output. Multi-level linear models, controlling for sex, sampling day, body mass index, and awakening time, revealed significant main effects of Age Group, Cortisol, and Age Group × Cortisol interactions on daily valence and affective choice responses. Higher daily cortisol was associated with lower average daily valence, particularly in younger compared to older adults. Similar patterns emerged for arousal, with an Age Group × Cortisol interaction indicating a stronger relationship in younger adults. These findings suggest that daily cortisol levels may have a weaker impact on emotional well-being with age.

